# Associations between life course socioeconomic circumstances and drinking patterns among young and early midlife Finnish public sector employees

**DOI:** 10.1093/alcalc/agag023

**Published:** 2026-04-24

**Authors:** Aino Salonsalmi, Eero Lahelma, Ossi Rahkonen, Anni Karjala, Anne Kouvonen, Tea Lallukka

**Affiliations:** Department of Public Health, University of Helsinki, P.O. Box 20, Tukholmankatu 8 B, 00014 University of Helsinki, Helsinki, Finland; Department of Public Health, University of Helsinki, P.O. Box 20, Tukholmankatu 8 B, 00014 University of Helsinki, Helsinki, Finland; Department of Public Health, University of Helsinki, P.O. Box 20, Tukholmankatu 8 B, 00014 University of Helsinki, Helsinki, Finland; Department of Public Health, University of Helsinki, P.O. Box 20, Tukholmankatu 8 B, 00014 University of Helsinki, Helsinki, Finland; Faculty of Social Sciences, University of Helsinki, P.O. Box 54, Unioninkatu 37, 00014 University of Helsinki, Helsinki, Finland; Centre for Public Health, Queen’s University Belfast, Institute of Clinical Science, Block A, Royal Victoria Hospital, Belfast BT12, 6BA, Northern Ireland; Department of Public Health, University of Helsinki, P.O. Box 20, Tukholmankatu 8 B, 00014 University of Helsinki, Helsinki, Finland

**Keywords:** alcohol drinking, alcohol drinking habits, socioeconomic factors, employee, cohort study, logistic models

## Abstract

**Aims:**

This study aims to examine associations between childhood and adulthood socioeconomic circumstances with drinking patterns.

**Methods:**

Data on socioeconomic circumstances and drinking patterns were derived from a survey among young and early midlife employees of the City of Helsinki in 2017 (*n* = 5875, 79% women). Logistic regression analysis was used to calculate odds ratios (ORs) and their 95% confidence intervals (CIs) for frequent, binge, and problem drinking (CAGE=Cutting down, Annoyance by criticism, Guilty feeling, Eye-openers). Childhood and adulthood socioeconomic circumstances were included. The models were adjusted for age and gender, and additionally for marital status and having underage children.

**Results:**

The mean alcohol units per week varied only little by socioeconomic circumstances although men with low material circumstances (6.3 units per week if low household wealth) drank somewhat more than their counterparts with better material circumstances (5.1 units per week if high household wealth). Binge drinking was more common among individuals with low socioeconomic circumstances, but frequent drinking among individuals with high socioeconomic circumstances. Differences in problem drinking varied by socioeconomic indicator. Adjusting for marital status and underage children attenuated the associations especially for binge and problem drinking. No socioeconomic measure was paramount although for binge and problem drinking household income and further material circumstances showed the strongest associations.

**Conclusions:**

There were only small differences by socioeconomic circumstances in the amount of alcohol used but individuals with low material circumstances most often showed adverse drinking patterns. Preventive measures focused on people with low material circumstances might diminish alcohol-related inequalities in health.

## Introduction

Alcohol use is a major burden to health and well-being worldwide, and accounts for 5.3% of all deaths (Shield et al. 2020). In work life, alcohol is associated for example with reduced productivity (Thørrisen et al. 2019), increased sickness absence (Marzan et al. 2022) and disability pension (Böckerman et al. 2016). People with low socioeconomic position experience greater alcohol-related harm than their higher position counterparts (Jones et al. 2015, Katikireddi et al. 2017). Alcohol use explains up to 27% of socioeconomic inequalities in mortality (Probst et al. 2020).

A systematic review found that people with high socioeconomic position consumed as much or even more alcohol than people with low socioeconomic position (Collins 2016). The discrepancy between the amount of alcohol consumed and alcohol-related harm is called the alcohol harm paradox (Probst et al. 2020; Boyd et al. 2022). Drinking patterns contribute to the harm incurred by alcohol drinking (Rehm et al. 2017) and different drinking patterns across socioeconomic groups partly explain the paradox (Thern and Landberg 2021; Boyd et al. 2022). Some previous studies have shown that binge drinking is more common among people with low socioeconomic position (Thern and Landberg 2021), but some others not (Collins 2016). High socioeconomic position has been associated with a reduced risk of alcohol use disorder (Calling et al. 2019). In Finland, people with high socioeconomic position use alcohol more often than those with low socioeconomic position (Mäkelä and Warpenius 2024), whereas both non-drinking and binge drinking are more common among people with low socioeconomic position (Peña et al. 2017).

Most studies examining socioeconomic differences in alcohol consumption have used conventional measures of socioeconomic position namely education, occupational class, or income (Huckle et al. 2010, Peña et al. 2017, Thern and Landberg 2021, Mäkelä and Warpenius 2024). Childhood socioeconomic position and further material circumstances in addition to income have been seldom included. Different socioeconomic measures portray different resources and exposures over the life course. Any single measure of socioeconomic position is unlikely to provide a comprehensive description of past and present socioeconomic circumstances (Lahelma and Rahkonen 2020). Education reflects non-material resources; occupational class contributes to the ranking in the society and to working conditions, while income portrays access to material resources. Despite decent income, individuals may have problems in paying bills and affording daily necessities (Wang et al. 2010). Thus, economic difficulties and further material conditions complement the conventional measures.

Studies on socioeconomic differences in drinking patterns with multiple measures of socioeconomic circumstances are largely lacking. A New Zealand study reported that education, occupational class, and income were all independently associated with drinking patterns with people with low socioeconomic circumstances drinking larger quantities and people with high socioeconomic circumstances drinking more frequently (Huckle et al. 2010). A UK study included employment status, education, occupational class and income, and home and car ownership (Beard et al. 2019). In general, individuals with low socioeconomic circumstances consumed alcohol less frequently but drank larger amounts and were more likely to report binge drinking (Beard et al. 2019). Occupational class and education were the strongest predictors of alcohol consumption followed by housing tenure (Beard et al. 2019). Another study reported higher rate of excess drinking but lower rate of binge drinking among owner-occupiers compared to those living in a rented accommodation (Fone et al. 2013). The association between childhood socioeconomic circumstances and later alcohol use has also been found (Collins 2016).

Age, cohort, alcohol policy, and other social and cultural factors contribute to drinking patterns. In Finland, the trend of alcohol consumption has mainly declined since 2007 in particular among young people (Mäkelä and Warpenius 2024). It is not known if socioeconomic differences in drinking patterns among young and early midlife adults remain the same after these changes. Marital status and having children are associated with both socioeconomic circumstances (Nisén et al. 2018, Karney 2021) and drinking patterns (Dinescu et al. 2017, Patrick et al. 2020) and should thus be considered when examining drinking patterns of young and early midlife adults. High alcohol consumption and problem drinking are associated with an increased risk of leaving the labour market and a lower chance of re-employment (Jørgensen et al. 2019). Thus, examining employees in the early phases of their work careers is important.

The primary aim of this study is to examine associations between childhood and adulthood socioeconomic circumstances and drinking patterns among young and early midlife employees. This is done by using multiple measures of socioeconomic position as well as drinking patterns. The secondary aim is to confirm if adjusting for marital status and underage children contributes to the associations.

## Materials and methods

The methods are reported following the STrengthening the Reporting of OBservational studies in Epidemiology guidelines (Benchimol and Langan 2025).

### Study design

The study was a cross-sectional study.

### Setting

The study was conducted among young and early midlife employees of the City of Helsinki, Finland in autumn 2017. The data were collected online and mail questionnaires, and an additional telephone survey was conducted among those who did not otherwise respond. The details of the data collection have been previously published (Lallukka et al. 2020).

### Participants

The employees of the City of Helsinki who were born in 1978 or later and with a job contract of at least 50% of regular work hours per week having lasted at least four months before the start of the data collection in 2017 (Lallukka et al. 2020). The City of Helsinki is the largest employer in Finland with nearly 38 000 employees annually in occupations ranging from manual workers to professionals and managers in various employment sectors (City of Helsinki 2023).

### Variables

#### Measures of socioeconomic circumstances

Measures of socioeconomic circumstances were used as exposure variables. Childhood socioeconomic circumstances consisted of parental education and childhood economic difficulties. The highest educational level of each parent was inquired by a four-level scale. The highest one of whichever parent was chosen to form a three-categorical variable: ‘Low’ included lower secondary education or lower, ‘intermediate’ vocational education or matriculation examination, and ‘high’ associate or academic degree. Childhood economic difficulties were dichotomized as ‘yes’ or ‘no’ based on a single-item question inquiring whether the respondent’s childhood family had considerable financial difficulties before the participant turned 16.

Conventional socioeconomic measures included participant’s own education, occupational class, and household income. The highest educational level was divided into ‘low’ (lower secondary education, vocational education or matriculation examination), ‘intermediate’ (bachelor’s degree), and high (master’s degree or higher). Low occupational class included manual and routine non-manual workers, intermediate occupational class semi-professionals and high occupational class professionals and managers. Monthly household disposable income was divided by household size and weighted according to the modified Organisation for Economic Co-operation and Development (OECD) equivalence scale. The participant received the value of 1.0, other adults of the household 0.5 and each child 0.3 (Hagenaars et al. 1994). The income groups were then divided into tertiles.

Further material resources were measured by current economic difficulties, housing tenure, and household wealth. The participants were inquired how often they had difficulties in paying bills and how often they had enough money to buy food or clothes for the participant or their family (Pearlin and Schooler 1978). Having either of these difficulties always, often, or sometimes implied current economic difficulties. Housing tenure was dichotomized into owner-occupiers and renters. Household wealth was divided into three groups: under €10 000; €10 000–99 999, and €100 000 or over.

#### Drinking patterns

Drinking patterns were used as outcome variables. Alcohol drinks per week were derived from the questions inquiring the participant’s current average consumption of beer or cider, wine, or mild beverages and spirits. Average consumption of alcohol per week was calculated with one drink equalling 12 g of pure ethanol.

Frequency of drinking was assessed by a question inquiring the participant’s current use of beer, wine, and spirits. There were 10 response alternatives from ‘I do not use alcohol’ to ‘daily or almost daily’ use. Women drinking once a week or more often and men drinking more often than once a week were classified as frequent drinkers.

Binge drinking was inquired by asking how often the respondent drank six or more alcohol units on a single occasion ranging from ‘never’ to ‘daily or almost daily’. Women binge drinking once a month or more often and men binge drinking more often than once a month were classified as binge drinkers.

Problem drinking was measured by the CAGE scale (Dhalla and Kopec 2007), including questions: Have you ever thought about cutting (C) down your drinking? Have you been annoyed (A) by criticism because of your drinking? Have your ever felt guilty (G) because of your drinking? Have you ever needed an eye-opener (E)? A positive answer to each question gives one point to a summary score ranging from zero to four. Among women the usual cut-point of two was used to indicate problem drinking. Among men the cut-point was set to three points.

#### Covariates

Gender (women/men) was derived from the questionnaire and a small number of missing responses (*n* = 18) were completed from the employee register for those with an informed consent to link survey and register data. Gender was included as a covariate. Age included three groups: 19 to 29, 30 to 34, and 35 years or over. Marital status categories were: ‘Single’, ‘married or co-habiting,’, and ‘divorced or widowed’. Underage children living at home was dichotomized (yes/no).

### Data sources

Data on all variables were retrieved from the questionnaire with the exceptions for occupational class, which was retrieved from the employer’s personnel register and for missing responses of gender, which were completed from the same register for those with an informed consent to linkage.

### Bias

According to the non-response analyses, the participants represent the target population quite well although individuals in low socioeconomic positions and with long-term sickness absence participated somewhat less (Lallukka et al. 2020). Other potential biases are considered in the discussion.

### Study size

**Figure 1:**
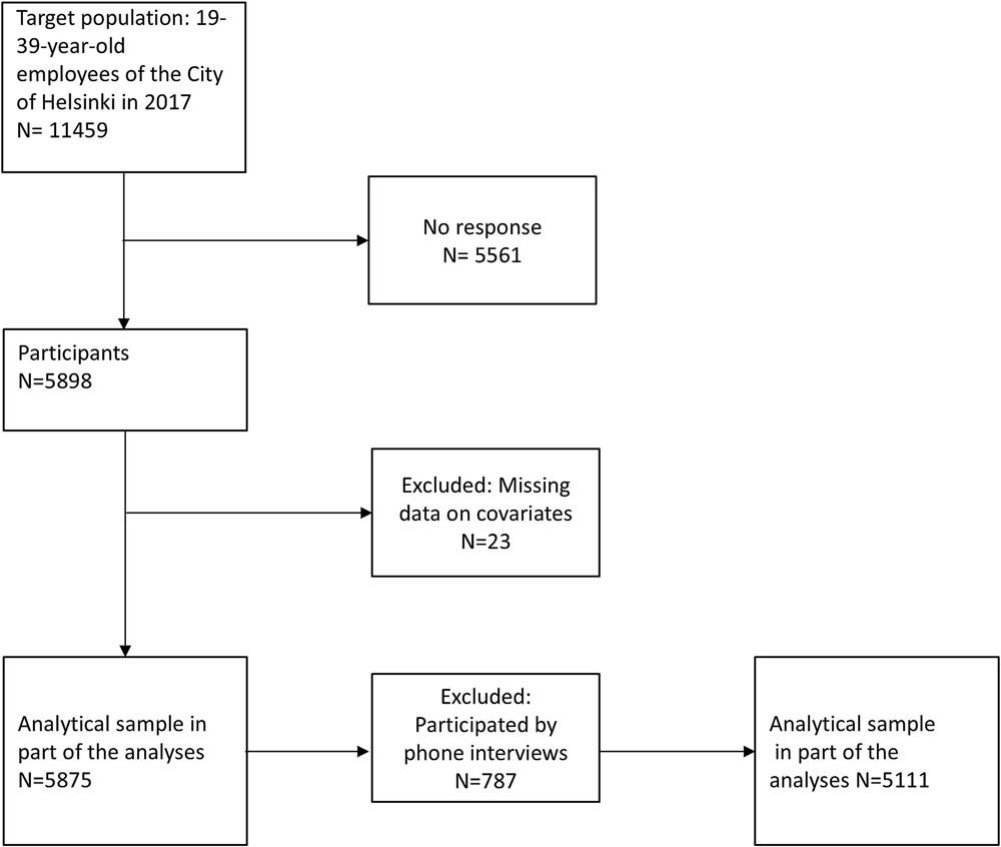
Flow-chat of the selection of the analytical sample of the study.

The target population was 11 459 employees and 5898 of them participated. The response rate was thus 51.5%. Most of the participants (80%) were women, corresponding to the gender distribution of the Finnish municipal sector. The telephone interview (*n* = 787) included less questions than the original questionnaire. Regarding alcohol variables only frequency of drinking and regarding socioeconomic circumstances only education was included. Data on occupational class were retrieved from employer’s personnel register among those who consented for the linkage. Thus, analyses on the frequency of drinking and education and occupational class included more participants than the other analyses since individuals who answered by phone were also included. Altogether 23 participants had missing data on covariates and were excluded. The analytical sample was 5875 participants. There was some item-nonresponse regarding specific socioeconomic (range 0.3%–2.9%) and alcohol (range 3.6–5.4%) variables. The final numbers of participants in each analysis are presented in Table 4. Figure 1 presents the selection of the study participants.

#### Statistical methods

First, we calculated percentages for drinking patterns and socioeconomic indicators. Second, drinking variables and socioeconomic indicators were cross-tabulated with covariates and drinking variables with socioeconomic indicators. Chi squared tests were performed. Third, mean number of alcohol units per week by socioeconomic indicators was calculated. Fourth, the associations between socioeconomic indicators and drinking patterns were analysed by logistic regression. Non-frequent, non-binge, and non-problem drinkers served as reference categories. The results are presented as odds ratios and their 95% confidence intervals (95% CI). Model 1 adjusts for age and gender Model 2, additionally for marital status and underage children living at home. As an exception, household income was not adjusted for marital status and underage children since the measure already considered other members living in the same household.

Women and men were pooled in the analyses with the exception for mean number of drinks per week. Interactions for gender in the logistic regression models were tested and statistically significant interactions were found only for the association between occupational class and frequent drinking and for the association between education and problem drinking. In addition, stratified analyses by gender were performed and for frequent drinking women with higher occupational class showed lower odds ratios compared to men.

Participants with missing data were omitted from the analyses. The final numbers of participants in each analysis are presented in Table 4.

We checked correlation analyses (Spearman’s correlation) for measures of socioeconomic circumstances. A strong correlation between education and occupational position (*r* = 0.79) and moderate correlations between household income and household wealth (*r* = 0.40) were observed. Otherwise, the correlations were markedly lower.

The analyses were also run omitting participants who did not work at the time of the survey (e.g. due to parental leave or long-term sickness absence), but the associations were practically similar and thus these participants were included in the analyses.

The SAS statistical package version 9.4 was used for the analyses.

### Ethical consideration

The Helsinki Health Study protocol was approved by the Ethics Committee of the Faculty of Medicine, University of Helsinki, and the City of Helsinki health and personnel authorities. The data protection statement is available from the Helsinki Health Study website: https://www.helsinki.fi/en/researchgroups/helsinki-health-study/data-protection-statement.

## Results

Over fifth of the participants were frequent drinkers and under fifth were binge and problem drinkers (Table 1). Both high and intermediate parental education were common, whereas low parental education was rare. Around fifth of the participants had experienced childhood economic difficulties. Of the participants, 58% lived in rented housing and around 22% reported current economic difficulties.

**Table 1:** Distributions of covariates, drinking patterns, and socioeconomic circumstances among the Helsinki Health Study participants in 2017 (*n*, %).

	*n*	%
**Gender**		
Women	4613	78.5%
Men	1262	21.5%
**Age**		
19–29	1864	31.7
30–34	1995	34.0
35 or over	2016	34.3
**Marital status**		
Single	1729	29.4
Married or cohabiting	3887	66.2
Divorced or widowed	259	4.4
**Underage children living at home**		
No	3787	64.5
Yes	2088	35.5
**Frequent drinking**		
Yes	1256	22.2
No	4410	77.8
**Binge drinking**		
Yes	892	18.3
No	3997	81.8
**Problem drinking**		
Yes	853	17.7
No	3959	82.3
**Parental education**		
High	2184	43.1
Intermediate	2417	47.7
Low	471	9.3
**Childhood economic difficulties**		
No	3879	78.4
Yes	1069	21.6
**Education**		
High	1679	28.6
Intermediate	2085	35.6
Low	2101	35.8
**Occupational class**		
High	1536	26.9
Intermediate	2225	39.0
Low	1942	34.1
**Household income**		
High	1910	37.7
Intermediate	1466	29.0
Low	1685	33.3
**Housing tenure**		
Owner	2161	42.6
Renter	2918	57.5
**Household wealth**		
High	1205	24.3
Intermediate	1999	40.4
Low	1746	35.3
**Current economic difficulties**		
No	3937	77.6
Yes	1138	22.4

Men drank on average 5.5 and women 2.4 units per week (Table 2). Problem drinking was more common among men compared to women. The participants under 30 years were more often binge drinkers whereas among older participants frequent drinking was more common. Those who were not married or cohabiting consumed more drinks per week and were more often binge or problem drinkers, whereas frequent drinking was most common among married or cohabiting participants. Participants who had underage children consumed less drinks per week and were less often binge or problem drinkers compared to the participants who did not have underage children.

**Table 2:** Covariates by drinking patterns. Proportions of Helsinki Health Study participants with specific drinking patterns (%).

	Drinks/week (s.d.)	Frequent drinking	Binge drinking	Problem drinking
**Gender**				
Men	5.5 (6.5)	20.8	18.8	21.9
Women	2.4 (2.9)	22.5	16.0	16.7
Chi-squared		0.200	0.043	0.000
**Age**				
19–29	3.1 (4.2)	16.7	25.3	18.4
30–34	3.0 (3.9)	23.0	17.0	17.6
35 or older	3.0 (4.3)	26.4	12.9	17.2
Chi-squared		<.0001	<.0001	0.654
**Marital status**				
Single	3.6 (5.2)	19.6	29.1	21.1
Cohabiting or married	2.8 (3.6)	23.3	13.4	16.3
Divorced or widowed	2.8 (3.6)	21.2	20.8	17.0
Chi-squared		0.010	<.0001	0.000
**Underage children at home**				
No	3.5 (4.6)	22.1	25.3	20.7
Yes	2.4 (3.2)	22.3	8.2	13.5
Chi-squared		0.836	<.0001	<.0001

Table 3 shows the indicators of socioeconomic circumstances by covariates. In general, low socioeconomic circumstances were associated with lower age with the exception of older age being associated with lower parental education and childhood economic difficulties. High socioeconomic circumstances were mostly associated with being married or cohabiting. The association between socioeconomic circumstances and underage children varied by socioeconomic indicator. Supplementary Table S1 presents the distributions of drinking patterns by measures of socioeconomic circumstances.

**Table 3:** Covariates by socioeconomic circumstances (%) among the Helsinki Health Study participants in 2017 survey.

	Gender		Age			Marital status			Underage children living at home	
	Women	Men	19–29	30–34	35+	Single	Married or cohabiting	Divorced or widowed	No	Yes
**Parental education**										
High	79	21	35	35	30	29	67	4	63	37
Intermediate	81	19	31	34	35	29	66	2	57	43
Low	79	21	21	30	50	27	65	8	49	51
Chi-squared		0.125			<.0001			0.008		<.0001
**Childhood economic difficulties**										
No	80	20	34	33	33	66	30	4	55	45
Yes	78	22	26	36	38	67	27	6	60	40
Chi-squared		0.145			<.0001			0.016		0.001
**Occupational class**										
High	77	23	18	39	43	23	73	4	61	39
Intermediate	84	16	35	35	31	30	66	4	63	37
Low	74	26	39	30	31	34	61	5	66	34
Chi-squared		<.0001			<.0001			<.0001		0.020
**Education**										
High	80	20	16	39	44	24	73	3	62	38
Intermediate	83	17	33	36	31	27	68	5	64	36
Low	73	27	43	28	29	36	59	5	67	33
Chi-squared		<.0001			<.0001			<.0001		0.003
**Household income**										
High	80	20	21	36	43	5	94	1	46	55
Intermediate	77	23	34	34	32	17	79	4	58	42
Low	81	19	42	32	26	67	23	9	75	25
Chi-squared		0.018			<.0001			<.0001		<.0001
**Housing tenure**										
Owner	80	20	17	36	46	14	83	3	44	56
Renter	80	20	43	32	25	41	54	6	70	30
Chi-squared		0.807			<.0001			<.0001		<.0001
**Household wealth**										
High	80	21	17	32	51	12	86	2	41	59
Intermediate	77	23	31	38	32	26	69	4	62	38
Low	83	17	43	32	25	44	49	6	68	32
Chi-squared		<.0001			<.0001			<.0001		<.0001
**Current economic difficulties**										
No	80	20	32	35	33	27	69	4	61	39
Yes	80	20	31	31	39	35	57	8	52	48
Chi-squared		0.944			0.0015			<.0001		<.0001

Among women, the variation of average number of drinks per week by socioeconomic indicators was small (Table 4). Men with low material circumstances consumed more drinks per week compared to men with high material socioeconomic circumstances. All studied socioeconomic measures were associated with frequent drinking among individuals with lower socioeconomic circumstances showing a decreased odds ratio of frequent drinking. Participants with low education (OR 0.33, 95% CI 0.28–0.39) and with low occupational class (0.39, 0.33–0.46) showed the lowest odds ratios for frequent drinking even after adjusting for marital status and underaged children.

**Table 4:** The associations between socioeconomic circumstances and drinking patterns among the Helsinki Health Study participants in 2017 survey. Odds ratios and their 95% confidence intervals from the logistic regression analyses.

	Drinks/week (s.d.)		Frequent drinking		Binge drinking		Problem drinking	
	Women	Men	Model 1	Model 2	Model 1	Model 2	Model 1	Model 2
**Parental education**								
*n*	3886	996	4869		4878		4804	
High	2.3 (3.0)	5.9 (6.5)	1.00	1.00	1.00	1.00	1.00	1.00
Intermediate	2.3 (2.8)	4.9 (6.0)	0.63 (0.55–0.73)	0.64 (0.56–0.73)	1.00 (0.86–1.17)	1.03 (0.88–1.21)	0.80 (0.68–0.93)	0.81 (0.69–0.94)
Low	2.3 (3.0)	5.9 (6.7)	0.59 (0.45–0.75)	0.60 (0.47–0.78)	1.12 (0.85–1.46)	1.17 (0.89–1.54)	0.85 (0.65–1.12)	0.87 (0.66–1.14)
**Childhood economic difficulties**								
*n*	3783	974	4747		4752		4681	
No	2.4 (2.8)	5.7 (6.6)	1.00	1.00	1.00	1.00	1.00	1.00
Yes	2.4 (3.2)	5.2 (6.5)	0.78 (0.66–0.92)	0.78 (0.66–0.93)	1.13 (0.95–1.35)	1.17 (0.97–1.40)	1.38 (1.16–1.65)	1.41 (1.18–1.68)
**Education**								
*n*	3889	998	5656		4883		4806	
High	2.5 (2.6)	5.0 (5.0)	1.00	1.00	1.00	1.00	1.00	1.00
Intermediate	2.4 (3.0)	5.7 (6.2)	0.52 (0.45–0.61)	0.53 (0.45–0.61)	1.26 (1.04–1.53)	1.32 (1.08–1.61)	0.85 (0.71–1.02)	0.86 (0.72–1.03)
Low	2.3 (3.1)	5.7 (7.5)	0.33 (0.28–0.39)	0.34 (0.29–0.40)	1.42 (1.16–1.72)	1.47 (1.20–1.80)	0.78 (0.64–0.94)	0.79 (0.65–0.95)
**Occupational class**								
*n*	3874	990	5497		4861		4785	
High	2.6 (2.9)	5.2 (5.5)	1.00	1.00	1.00	1.00	1.00	1.00
Intermediate	2.3 (2.9)							
Low	2.3 (3.0)	5.8 (6.1)	0.58 (0.50–0.68)	0.59 (0.51–0.69)	1.23 (1.02–1.49)	1.27 (1.04–1.55)	0.82 (0.68–0.98)	0.82 (0.68–0.99)
**Household income**		5.5 (7.4)	0.39 (0.33–0.46)	0.40 (0.34–0.48)	1.31 (1.07–1.60)	1.32 (1.07–1.62)	0.80 (0.66–0.97)	0.80 (0.66–0.97)
*n*	3872	996	4855		4866		4787	
High	2.4 (2.9)	5.2 (5.3)	1.00		1.00		1.00	
Intermediate	2.2 (2.6)	5.2 (6.3)	0.76 (0.64–0.89)		1.33 (1.09–1.61)		1.20 (1.00–1.45)	
Low	2.5 (3.2)	6.3 (8.0)	0.63 (0.53–0.74)		2.01 (1.67–2.40)		1.31 (1.10–1.58)	
**Housing tenure**								
*n*	3884	999	4871		4879		4802	
Owner	2.3 (2.8)	5.1 (5.5)	1.00	1.00	1.00	1.00	1.00	1.00
Renter	2.4 (3.0)	5.9 (7.2)	0.80 (0.69–0.91)	0.79 (0.68–0.91)	1.63 (1.38–1.91)	1.21 (1.02–1.43)	1.23 (1.05–1.44)	1.10 (0.93–1.30)
**Household wealth**								
*n*	3782	986	4755		4764		4694	
High	2.4 (3.1)	5.1 (5.4)	1.00	1.00	1.00	1.00	1.00	1.00
Intermediate	2.3 (2.5)	5.3 (6.5)	0.83 (0.71–0.99)	0.81 (0.68–0.96)	1.42 (1.14–1.77)	1.15 (0.92–1.44)	1.10 (0.90–1.35)	1.01 (0.82–1.24)
Low	2.5 (3.2)	6.3 (7.5)	0.73 (0.61–0.87)	0.74 (0.61–0.89)	2.26 (1.82–2.81)	1.66(1.32–2.08)	1.44 (1.17–1.77)	1.29 (1.04–1.60)
**Current economic difficulties**								
*n*	3882	998	4867		4876		4800	
No	2.3 (2.8)	5.4 (6.2)	1.00	1.00	1.00	1.00	1.00	1.00
Yes	2.6 (3.4)	6.1 (7.6)	0.69 (0.58–0.82)	0.73 (0.62–0.87)	1.44 (1.22–1.71)	1.46 (1.22–1.74)	1.29 (1.09–1.53)	1.32 (1.11–1.57)

Model 1 is adjusted for age and gender, Model 2 additionally for marital status and underage children living at home. Household wealth was not adjusted for marital status and underage children living at home since the measure already considered other members living in the same household.

Childhood socioeconomic indicators were not associated with binge drinking. All conventional socioeconomic measures were associated with binge drinking and participants with low socioeconomic circumstances showed increased odds ratios (Table 4). Low household income showed the highest odds ratio (2.01, 1.67–2.40). The associations concerning low education (1.47, 1.20–1.80) and low occupational class (1.32, 1.07–1.62) remained after adjustments for marital status and underage children. Also having low material socioeconomic circumstances was associated with an increased odds ratio of binge drinking. These associations remained when adjusting for marital status and for underage children (1.21, 1.02–1.43 for housing tenure, 1.46, 1.22–1.74 for current economic difficulties, 1.66, 1.32–2.08 for household wealth).

Intermediate parental education was associated with decreased problem drinking whereas childhood economic hardship was associated with an increased odds of problem drinking (Table 4). The associations remained when adjusting for marital status and underage children (0.81, 0.69–0.94 for intermediate parental education and 1.41, 1.18–1.68 for childhood economic hardship). Low education and low occupational class were associated with decreased problem drinking and the associations remained after adjusting for marital status and underage children (0.79, 0.65–0.95 for low education and 0.80, 0.66–0.97 for low occupational class). Low household income, rented housing, current economic difficulties, and low household wealth all showed an association with increased problem drinking. The association concerning current economic difficulties (1.32, 1.11–1.57) and low household wealth (1.29, 1.04–1.60) remained after adjusting for marital status and underage children.

## Discussion

This study examined the association between multiple childhood and adulthood socioeconomic circumstances and their associations with drinking patterns among young and early midlife public sector employees. The average number of drinks per week varied only little by socioeconomic circumstances although men with low material socioeconomic circumstances drank somewhat more compared to men with high socioeconomic circumstances. Frequent drinking was more common among individuals with high socioeconomic circumstances whilst binge drinking was more common among individuals with low socioeconomic circumstances. Concerning problem drinking the associations varied by indicators of socioeconomic circumstances.

The number of alcohol drinks per week was rather low among both women and men reflecting the decline in overall consumption of alcohol in Finland. Despite rather low overall alcohol consumption, binge and problem drinking were common, as in the latest national study (THL 2023). Thus, binge drinking is still important part of the Finnish drinking patterns. Men drank more compared to women and also frequent, binge drinking, and problem drinking were more common among men when considering the different cut-points between genders.

In line with previous research, there was little variation in the amount of alcohol consumed across socioeconomic circumstances (Collins 2016). Men with low household income and low other material socioeconomic circumstances, however, drank somewhat more compared to men with high socioeconomic circumstances. Other drinking patterns showed clear differences by socioeconomic circumstances. In line with previous studies (Peña et al. 2017, Thern and Landberg 2021), binge drinking was more common among individuals with low socioeconomic circumstances. In previous Finnish studies, the socioeconomic differences have varied when measured by less severe indicators of binge drinking (Paljärvi et al. 2013, Härkönen et al. 2017). In the present study clear differences were observed with a rather modest measure of binge drinking. Binge drinking is independently associated with somatic diseases, mental disorders, and accidents (Kuntsche et al. 2017, Rehm et al. 2017) and thus individuals with low socioeconomic circumstances are extra vulnerable to adverse effects of drinking at the same overall consumption level.

Individuals with high socioeconomic circumstances were more often frequent drinkers compared to those with low socioeconomic circumstances in accordance with previous studies (Collins 2016, Huckle et al. 2010, Beard et al. 2019, Mäkelä and Warpenius 2024). However, frequent drinking among people with high socioeconomic circumstances is not necessarily without harm, but it has been associated for example with lower fertility, cancer, and liver disease without safe limits (Fan et al. 2017, Rehm et al. 2017).

The associations between socioeconomic circumstances and problem drinking varied. In line with a previous Scottish study (Batty et al. 2008), low income and low further material circumstances were associated with increased problem drinking, whereas low education and low occupational class were associated with decreased problem drinking. These results are in contrast with previous evidence which has found both conventional and further material socioeconomic circumstances to be associated with problem drinking (Batty et al. 2008). Our results raise the question if individuals with high education and high occupational class were more likely to answer positively to the CAGE questions. This could be due to stricter own and surrounding attitudes toward alcohol drinking, since there were no major differences in the number of drinks per week, and binge drinking being less common among higher educational and occupational classes compared to individuals with low education and low occupational class. Previous studies have not reported that socioeconomic position might bias screening positive on the CAGE scale. Studies on socioeconomic differences in attitudes towards alcohol are scarce but low education has been associated with less supportive attitudes towards alcohol control measures (Reile and Rehm 2023).

Previous studies on childhood socioeconomic circumstances and alcohol drinking have often lacked clear associations (Wiles et al. 2007, Collins 2016). In line with some previous research (Batty et al. 2008) in the present study associations were, however, found. Low and intermediate parental education and no economic difficulties in childhood were associated with less frequent drinking. This might reflect parental drinking habits i.e. parents with high socioeconomic circumstances might have been frequent drinkers themselves. In addition, childhood economic difficulties were associated with problem drinking.

There were no major differences in the associations between conventional measures and further material socioeconomic circumstances with the exception for the previously discussed findings concerning problem drinking. However, men with low material socioeconomic circumstances drank somewhat more drinks per week and the associations between material socioeconomic circumstances and binge and problem drinking were particularly strong. This suggests that people with poorest material circumstances most often had adverse drinking patterns. High income and material possessions might enable individuals to more often engage in healthier behaviors and have such leisure time activities that do not involve alcohol drinking. Adverse drinking habits might affect the career and result in lower income and low household wealth. An earlier Scottish study found that car ownership and housing tenure were marginally more strongly related to heavy alcohol intake and problem drinking than more conventional socioeconomic measures (Batty et al. 2008). However, another UK study reported that occupational class, education, and housing tenure were the strongest socioeconomic predictors of alcohol consumption (Beard et al. 2019).

Being married or cohabiting and having underage children at home attenuated and partly abolished the associations between socioeconomic circumstances and binge and problem drinking whereas their contribution to frequent drinking was modest. Being married or cohabiting and having children were associated with high socioeconomic circumstances. Previous research has shown similar associations among men (Nisén et al. 2018). It might be that having a family decreases binge and problem drinking and shapes drinking patterns (Dinescu et al. 2017, Patrick et al. 2020) to favour frequent drinking of small amounts of alcohol. It is also possible that individuals with binge and problem drinking are more likely to remain without a partner or family.

The strengths of this study include large data and the possibility to use several measures of both socioeconomic circumstances and drinking patterns. Yet, people tend to underestimate their drinking (Stockwell et al. 2018). Childhood socioeconomic circumstances were measured retrospectively which might bias the results (Hardt and Rutter 2004). Non-response analyses suggest no major bias (Lallukka et al. 2020) but it is possible that people with the highest alcohol consumption and most adverse drinking patterns were self-selected to non-respondents (Stockwell et al. 2018). In addition, individuals likely with most adverse drinking habits were not included in the target population as the study only included people who were originally employed. Overall, the study included employed young and early midlife individuals in the public sector and thus the results might not fully apply to older workers, other employment sectors, or people outside the workforce. However, although the strengths of the associations could be assumed to vary in different populations, there is no particular reason to assume why the direction of the socioeconomic differences in drinking patterns would be different in this cohort as compared to other groups. Our results are in line with the findings from previous studies in other age groups and populations (Collins 2016, Thern and Landberg 2021).

Alcohol consumption in Finland has mainly declined during the past 15 years, but binge drinking remains frequent (Mäkelä and Warpenius 2024). The present study focused on employees and confirmed that these national findings apply for employed people. Alcohol-related harm is common in Finland and high alcohol consumption has been associated for example with sickness absence (Marzan et al. 2022) and disability retirement (Böckerman et al. 2016). It has been shown that preventive measures such as brief alcohol intervention can reduce alcohol consumption compared to minimal or no intervention (Kaner et al. 2018). Occupational health care covers almost all employees in Finland, irrespective of their socioeconomic position, and preventive actions against harmful drinking patterns should be carried out in pre-employment medical examinations and other contacts of occupational health services.

In conclusion, this study showed that despite declining trend in alcohol use in Finland, socioeconomic differences remain among young and early midlife public sector employees. For binge and problem drinking, household income and further material socioeconomic circumstances showed the strongest associations suggesting that people living in the most deprived socioeconomic circumstances were most likely to engage in adverse drinking patterns. Preventive measures are thus needed especially among those with low material socioeconomic circumstances to prevent future socioeconomic differences in health and work ability incurred by alcohol. In addition to national measures, occupational health care plays a crucial role in prevention.

## Supplementary Material

agag023_ALkosepsupplement2026

## Data Availability

The Helsinki Health Study survey data (and the register data of the City of Helsinki) cannot be made publicly available due to strict data protection laws and regulations. The data can only be used for scientific research. More information on the survey data can be requested from the Helsinki Health Study research group (kttl-hhs@helsinki.fi).
