# Supplementary material for: Associations between life course socioeconomic circumstances and drinking patterns among young and early midlife Finnish public sector employees
Source: Alcohol Alcohol. 2026 Apr 24;61(3):agag023. doi: 10.1093/alcalc/agag023 (PMC13107316; doi:10.1093/alcalc/agag023)
Supplement: agag023_ALkosepsupplement2026 [file agag023_alkosepsupplement2026.docx]

Supplemental table S1. The distributions of drinking patterns (n,%) by socioeconomic circumstances among the Helsinki Health Study participants. P-values from chi-squared test.

|  | Frequent drinking | | Binge drinking | | Problem drinking | |
| --- | --- | --- | --- | --- | --- | --- |
|  | No | Yes | No | Yes | No | Yes |
| **Parental education** |  |  |  |  |  |  |
| High | 1509, 72 | 594, 28 | 1717, 82 | 388, 18 | 1664, 80 | 406, 20 |
| Intermediate | 1848, 80 | 473, 20 | 1908, 82 | 420, 18 | 1923, 84 | 370, 16 |
| Low | 355, 80 | 90, 20 | 365, 82 | 80, 18 | 366, 83 | 75, 17 |
| Chi-squared |  | <.0001 |  | 0.937 |  | 0.010 |
| **Childhood economic hardship** |  |  |  |  |  |  |
| No | 2819, 76 | 910, 24 | 3066, 82 | 668, 18 | 3067, 83 | 607, 17 |
| Yes | 809, 79 | 209, 21 | 825, 81 | 193, 19 | 791, 79 | 216, 21 |
| Chi-squared |  | 0.010 |  | 0.432 |  | 0.000 |
| **Education** |  |  |  |  |  |  |
| High | 1069, 66 | 559, 34 | 1231, 86 | 202, 14 | 1137, 80 | 276, 20 |
| Intermediate | 1595, 79 | 415, 21 | 1447, 81 | 337, 19 | 1455, 83 | 304, 17 |
| Low | 1737, 86 | 281, 14 | 1313, 79 | 353, 21 | 1362, 83 | 272, 17 |
| Chi-squared |  | <.0001 |  | <.0001 |  | 0.095 |
| **Occupational class** |  |  |  |  |  |  |
| High | 997, 67 | 491, 33 | 1138, 85 | 195, 15 | 1055, 80 | 262, 20 |
| Intermediate | 1688, 79 | 457, 21 | 1568, 81 | 375, 19 | 1595, 83 | 323, 17 |
| Low | 1581, 85 | 283, 15 | 1264, 80 | 321, 20 | 1286, 83 | 264, 17 |
| Chi-squared |  | <.0001 |  | 0.000 |  | 0.056 |
| **Household income** |  |  |  |  |  |  |
| High | 1318, 71 | 536, 29 | 1623, 87 | 240, 13 | 1550, 84 | 287, 16 |
| Intermediate | 1089, 77 | 317, 23 | 1166, 83 | 247, 17 | 1129, 82 | 255, 18 |
| Low | 1291, 81 | 304, 19 | 1190, 75 | 400, 25 | 1258, 80 | 308, 20 |
| Chi-squared |  | <.0001 |  | <.0001 |  | 0.007 |
| **Housing tenure** |  |  |  |  |  |  |
| Owner | 1522, 73 | 570, 27 | 1828, 87 | 275, 13 | 1746, 84 | 332, 16 |
| Renter | 2189, 79 | 590, 21 | 2160, 78 | 616, 22 | 2204, 81 | 520, 19 |
| Chi-squared |  | <.0001 |  | <.0001 |  | 0.005 |
| **Household wealth** |  |  |  |  |  |  |
| High | 833, 72 | 332, 29 | 1039, 89 | 133, 11 | 974, 84 | 179, 16 |
| Intermediate | 1466, 76 | 456, 24 | 1604, 83 | 321, 17 | 1580, 83 | 324, 17 |
| Low | 1321, 79 | 347, 21 | 1243, 75 | 424, 25 | 1297, 79 | 340, 21 |
| Chi-squared |  | <.0001 |  | <.0001 |  | 0.001 |
| **Current economic difficulties** |  |  |  |  |  |  |
| No | 2823, 75 | 949, 25 | 3137, 83 | 645, 17 | 3096, 83 | 630, 17 |
| Yes | 886, 81 | 209, 19 | 849, 78 | 245, 22 | 852, 79 | 222, 21 |
| Chi-squared |  | <.0001 |  | <.0001 |  | 0.005 |
